# The Impact of Body Image on the WTP Values for Reduced-Fat and Low-Salt Content Potato Chips among Obese and Non-Obese Consumers

**DOI:** 10.3390/nu8120830

**Published:** 2016-12-21

**Authors:** Tiziana de-Magistris, Belinda López-Galán, Vincenzina Caputo

**Affiliations:** 1 Unidad de Economía Agroalimentaria, Centro de Investigación y Tecnología Agroalimentaria de Aragón, Instituto Agroalimentario de Aragón (IA2) (CITA-Universidad de Zaragoza), Avda Montañana 930, Zaragoza 50059, Spain; belindasusanlopez@gmail.com; 2 Agricultural, Food, and Resource Economics, Michigan State University, East Lansing, MI 48824, USA; vcaputo@anr.msu.edu

**Keywords:** body image, food, practice valuation and purchase, obesity, body mass index

## Abstract

The aim of this study is to assess the influence of body image on consumers’ willingness to pay (WTP) for potato chips carrying nutritional claims among obese and non-obese people. About 309 non-clinical individuals participated in a Real Choice Experiment. They were recruited by a company and grouped in: (i) non-obese with good body image; (ii) non-obese with body image dissatisfaction; (iii) obese with good body image; (iv) obese with body image dissatisfaction. Results indicate differences in consumers’ willingness to pay among consumer groups. Body image dissatisfaction of normal people did not influence the WTP for healthier chips. Obese people with body image dissatisfaction were willing to pay more for healthier chips (i.e., low-salt content potato chips) than normal ones with body image dissatisfaction. Examining the role of knowledge in the light of how this could impact on body image is relevant to improve the health status of individuals and their diet. Knowledge about nutrition could improve the body image of obese people.

## 1. Introduction

Nowadays the obesity epidemic is dramatically becoming a big issue due to the increasing rates of being overweight and obesity around the world. According to the International Association for the Study of Obesity, the prevalence of obesity (BMI > 30 kg/m^2^) in many European countries has exceeded 20%. In particular, it is estimated in 2014 that in the EU-27, over 53% of the EU population is either overweight or obese, as around 34.2% of adults are overweight and 13.7% are obese [1].

Therefore, these people are subject to several forms of social injustice and unfair treatment. In their review paper, Puhl and Heuer [2] summarize weight discrimination in two different categories of settings. The first one involves unfair treatment in employment, health care, and educational settings. For example, people who are obese are less likely to be hired for a job, receive a promotion, and more likely to experience wrongful termination, because they are considered to have low willpower by health-care professionals and a lower chance of attaining higher education. In this regard, recently Gupta et al. [3] demonstrated that obese people are more likely to present more absenteeism than people of normal weight. The authors reported that between 6.5% and 7.9% of obese people have been absent due to weight-related health problems. In addition, the cost of absenteeism in obese people was higher than €1000 per worker per year compared to €896 per worker per year generated by normal-weight people.

The second kind of discrimination has a psychological nature since it can impact the psychological health and well-being of those who suffer obesity. For example, it has been demonstrated that this discrimination contributes to depression, anxiety, and low self-esteem, and influences the perception that obese people have about their own body. This perception is called “body image” and has been studied by Thomas Cash and colleagues [4]. To measure this perception, Cash and his colleagues proposed the Body Image State Scale (BISS), which classifies people into two groups: people with good body image (self-accepting) and people with body image dissatisfaction. Currently, the empirical evidence has shown that people with body image dissatisfaction could restrict or not restrict their eating behavior, and also indicated that good body image was adversely influenced by the consumption of high-calorie food. For example, Lattimore, Walton, Bartlett, Hackett, and Stevenson [5] revealed that body image dissatisfaction was associated with higher BMI and dietary restraint among women. In the same line, Vocks, Legenbauer, and Heil [6] also found that good body image was adversely influenced by the consumption of high-calorie food. Nevertheless, Milkewicz and Cash [7] indicated that body image dissatisfaction and women’s binge eating were significantly correlated. Finally, Cash, Melnyk, and Hrabosky [8] found that body image dissatisfaction was positively related to eating disturbances while Cash and Fleming [9] showed that body image dissatisfaction impacted negatively the ability to control their weight. Although the mentioned studies contribute important information to the literature about the level of body image as a factor in the development of healthy or unhealthy eating behavior, none of them reported quantitative and economic evidence of the impact of body image dissatisfaction on purchase behavior. Since there remain significant gaps concerning this relationship, our study aims to fill this gap in the literature. 

Therefore, the objective of this study is to test whether the perception of body image of obese and non-obese consumers would affect willingness to pay (WTP) for potato chips carrying nutritional claims (reduced-fat and low-salt). In order to achieve the objective of this study, we conducted a Real Choice Experiment (RCE) which is the most widely used stated preference multi-attribute method for valuing products or attributes. 

In this study, we recruited a non-clinical sample of participants (i.e., obese and normal-weight consumers who are not under any clinical treatment) who revealed their WTP for potato chips which carry some nutritional claims. In this regard, it is widely recognized that nutritional claims could be considered an important policy tool to help individuals to make healthier food choices [10]. Moreover, the results of the present study are expressed in terms of willingness to pay in euros which is a quantifiable measure since we examine the impact of body image dissatisfaction when people are shopping rather than consuming food. These are the main contributions of this study. Quantifying the body image phenomenon is quite important because of its implications. To illustrate, if we find differences in preferences and WTP between obese and normal weight people we may be able to affirm that the body image dissatisfaction of obese people affects not only eating behavior but also purchase behavior towards healthier food products. 

Currently, understanding the predictors of purchase behavior for people with body image dissatisfaction is thus critical in light of the negative consequences associated with unhealthy food choice in terms of physical health. Hence, the communication and promotion campaigns designed by governments and food companies about food products and habits can take into account this psychological aspect of obese consumers. Indeed, our results present important food policy implications given that people who are obese are more likely to be vulnerable to unhealthy eating patterns, unsuccessful dieting and weight cycling, and making unhealthy food choices. Finally, to the best of our knowledge, this is the first study which assesses the effect of body image on WTP values revealed through real choice experiment (RCE) among people who are obese and those who are not. 

## 2. Materials and Methods 

### 2.1. Recruitment and RCE Procedures 

In our study, we used the RCE since a well-known shortcoming of the stated preference CE approach is hypothetical bias, defined as the difference between values obtained through hypothetical methods and the values (or what an individual might actually pay for the provision of the goods) obtained through non-hypothetical methods [11,12]. To mitigate this bias, several researchers in the CE literature have started using the so-called non-hypothetical or Real Choice Experiment (RCE), which incorporates both an incentive-compatible mechanism and real products [13,14,15,16,17]. The interpretation of these findings is that WTP values from RCE can be assumed to be the true values corresponding to actual payments in the marketplace.

The experiment was conducted in the capital town of a Spanish region in the period March–May 2015. Participants were randomly recruited by a subcontracted professional market research agency using a stratified sampling procedure, by gender, age, and BMI. The company actively recruited respondents in the population and the experiment was conducted on the premises of the company. The target population of our study was primary food buyers in households, households who consumed potato chips, and consumers that were at least 18 years old. 

**Figure 1:**
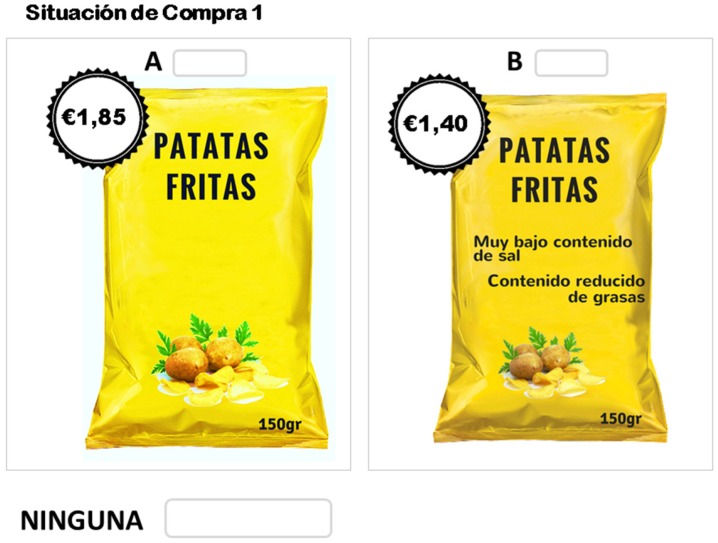
Example of a choice set.

A total of 309 individuals participated in our RCE in groups with a maximum of 10–12 people, seated separately and far from each other to avoid communication between them during the experiment. All subjects gave their informed consent for inclusion before they participated in the study. The study was conducted in accordance with the Declaration of Helsinki, and the protocol was approved by the Ethics Committee of CITA (FP7-MC-CIG-332769). The RCE was conducted as follows. Before beginning the experiment, participants completed a questionnaire and they received €10 in cash at the end of the session as a participation fee. The questionnaire was designed to measure participants’ body image. In their seminar paper, Cash, Fleming, Alindogan, Steadman, and Whitehead [4] designed a new scale called the Body Image State Scale (BISS) that measured current body experience, used for both sexes and in any specific time or context. In this regard, the Body Image State Scale (BISS) assesses six fields: overall physical appearance; body size and shape; weight feelings; physical attractiveness; current feelings about one’s looks compared to how one typically feels; and the evaluation of one’s own appearance compared to the average person’s appearance. Items were rated on a nine-point, bipolar, Likert scale. Based on their BISS mode and BMI, subjects were then allocated into four different groups as follows: (i) non-obese people (BMI less than 30 kg/m^2^) with good body image (BISS more than 30) (NH); (ii) non-obese people (BMI less than 30 kg/m^2^) with body image dissatisfaction (BISS less than 30) (NL); (iii) obese people (BMI more than 30 kg/m^2^) with good body image (BISS more than 30) (OH); (iv) obese people (BMI more than 30 kg/m^2^) with body image dissatisfaction (BISS < 30) (OL). In addition, participants were informed previously about the product, the methodology, and the objective of the research. They were allowed to inspect the different potato chips on the market, containing nutritional facts in the choice sets. However, they did not receive any information about brand, ingredients, and processing method of the potato chips. Then, they were also told that they would be faced with different choice tasks, each described by three choice options: two different potato chips and a no-purchase option (see Figure 1). For each of these choice sets, they were asked to select on a sheet of paper the alternatives in each choice task they wanted to buy, if any. Finally, at the end of the experiment, each participant drew a number from an envelope between 1 and 12 (total number of choice sets), to determine the binding choice set. Accordingly, participants had to purchase and pay the ‘posted price’ for the potato chips they picked, if any, in the binding choice set. Participants received the packet of potato chips after paying for the product they chose, unless they picked the no-purchase option in the binding choice set. 

### 2.2. Product and Choice Experiment Design

In this study, we chose potato chips as the product of interest. This is because potato chips are a high-density product that may evoke a hedonic pleasure and different responses from obese and non-obese participants due to an associated cue (e.g., smell) [18]. For instance, some studies reported positive associations between cravings for high-density food and body mass index [19]. Table 1 shows the selected attributes and corresponding levels used in the RCE. To mirror the range of current market prices in Spanish supermarkets for a packet of potato chips (150 g), four price levels were considered. These are: €0.50, €0.95, €1.40 and €1.85. The second attribute was a reduced-fat content claim and the third attribute was a low-salt content claim. We selected reduced-fat content claims because it is scientifically proven that the increase in energy intake from fats is one of the factors influencing the prevalence of obesity worldwide, and that a diet with low levels of fats is more effective for those patients with obesity who are trying to control levels of lipoprotein cholesterol (LDL) [20,21]. Some studies have also indicated an association between the prevalence of salt consumption and an increased risk of osteoporosis, kidney disease, obesity, stomach cancer, and increased blood pressure [22,23,24]. The World Health Organization (WHO) recommends not exceeding 5 g/day consumption of salt [25].

We defined two levels for reduced-fat content: unlabeled (conventional or a packet of chips that did not carry the EU nutritional claim) and reduced-fat claim (a packet of chips that carried the EU nutritional claim). We defined two levels of low-salt claims. The first level corresponded with a packet of chips without a label indicating a low level of salt content. The second level corresponded with a nutritional claim indicating that the chips were produced with 0.30 g of salt per 100 g of chips. The information on the attributes and the nutritional claims was clearly explained to participants and the translation from Spanish is shown in Table 1. 

Estimation accuracy can be increased at given sample sizes by adopting a sequential experimental design that progressively and iteratively optimises some efficiency criterion [26]. Following Scarpa et al. [27], the choice tasks were designed using a sequential Bayesian approach to minimize the D-error. The sequential Bayesian approach was performed in three steps. In the pilot, the design was derived assuming multinomial probability specification. Hence, the selected attributes and their level were used to come up with an orthogonal factorial design. Data from the pilot study was used to estimate a model whose coefficient estimates were then used as Bayesian priors. The design consisted of 12 choice tasks where each choice set included three alternatives: two designed alternatives consisting of different products, and a no-purchase scenario. The choice design was obtained using Ngene software version 1.1.2 [28].

### 2.3. Measures: Model Specification 

Choice experiments are consistent with the Random Utility theory [29] and Lancaster theory [30] of consumer demand. Given the attribute and attribute levels selected in this study to describe potato chips, the utility that an individual *n* derives from a product alternative *j* at choice occasion *t* can be derived as follows:

(1) $$U_{\mathrm{njt}}=NOBUY+\beta_{1}PRICE_{njt}+\beta_{2}FAT_{njt}+\beta_{3}SALT_{njt}+\beta_{4}FSAL_{njt}+\varepsilon_{\mathrm{njt}}$$

NOBUY is the alternative-specific constant, coded as a dummy variable equal to 1 for the non-purchase option and 0 otherwise. The price (*PRICE*) variable enters into the model as a continuous variable. The nutritional claims labels *FAT*, *SALT*, and *FSALT*, which represent the interaction between the reduced-fat and salt claims are coded as dummy variables because they indicate whether the corresponding claims analyzed are present or absent in the model. Finally, $\varepsilon_{\mathrm{njt}}$ is an unobserved random term that is distributed following an extreme value type I (Gumbel) distribution, independent and identically distributed (i.i.d.) over alternatives and independent of *β*. We used the random parameters logit (RPL) model to estimate consumer preferences for potato chips with reduced-fat and low-salt claims. This model assumes heterogeneous preferences around the mean parameters through the estimation of standard deviations associated with each random parameter estimate [31,32].

Given the fact the participants were grouped in four different groups, we used a test of the joint equality for the estimated parameters to test whether estimates from the RPL were equivalent across the four groups. The test for equality is −2 (LLj − ΣLLi) which is distributed χ^2^ with K × (M − 1) degrees of freedom, where LLj is the log likelihood value for the pooled data (all four groups), LLi are the log likelihood values for the different restricted models (groups), K is the number of parameters, and M is the number of groups [33]. The null hypothesis of the test is that the parameters of the RPL models are equal across the four groups. If this hypothesis is rejected, we are able to compare the estimated WTP values among the groups because the error variance is constant within each group and it will be cancelled out in the calculation of the marginal WTPs.

Based on the estimated coefficients from Equation (1) we calculated the mean marginal WTP values for each attribute by taking the ratio of the mean parameter estimated for the non-monetary attributes to the mean price parameter multiplied by minus one. Then, we used the combinatorial test suggested by Poe, Giraud, and Loomis [34] in order to compare differences between estimated mean WTP in the four different groups. This non-parametric test first requires the generation of a distribution of 1000 WTP estimates using, for example, the parametric bootstrapping method proposed by Krinsky and Robb [35]. 

## 3. Results

Table 2 reports the socio-demographic characteristics of the participants in the four groups. As can be noted, the majority of normal weight people with good body image (NH) were young women less than 35 years old with a university degree. However, the majority of normal weight people with body image dissatisfaction (NL) were older women (between 35–55 years old) with secondary level of education. Nevertheless, the majority of obese people with good body image were men and older than 55 years with a secondary level of education. Finally, obese people with body image dissatisfaction were mostly represented by women, with an age between 35–55 years and secondary level of education. 

Table 3 reports the likelihood values for the pooled sample and each group together with the tests of equality for the RPL model. The results indicate that the joint null hypotheses of equality between the four groups (LR = 54.9) are rejected, suggesting that it would be appropriate to compare the estimated WTPs across the four groups.

Moreover, Table 3 presents the coefficient estimates from the RPL model across the different consumer groups. As expected, in all groups, the standard deviations for the random variables are statistically significant, indicating heterogeneity in consumer preferences for the reduced-saturated fat and salt content claims. Also, the alternative-specific constant (NOBUY) is negative and significant, indicating that consumers gained a lower utility from the no-purchase option than for the buy alternatives. Moreover, as expected, the price variable (PRICE) is negative and statistically significant in accordance with economic theory. Given the main objective of this study as well as potential differences in scales across consumer groups [36], we interpreted the results associated with consumer valuation for both health claims in the context of willingness to pay estimates.

**Figure 2:**
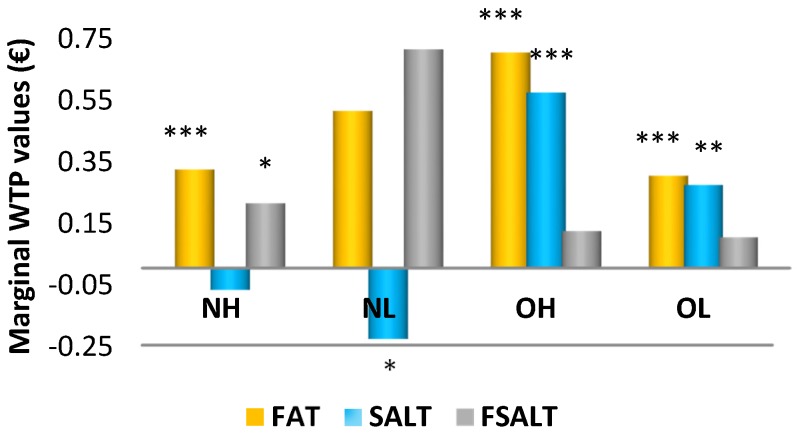
Marginal WTP values (€/150 g) across four groups. (***), (**), (*) denotes statistical significance at 1%, 5%, and 10% significance.

Table 4 shows the statistical significance from the Poe Test across four groups and Figure 2 reports the marginal WTP estimates across the different consumer groups. In the NH group, consumers were willing to pay a premium price for potato chips carrying a reduced-fat claim and for both reduced-fat and low-salt claims when they appeared jointly. Consumers who belonged to the NL group valued the low-salt claim negatively. Finally, when comparing the NH and NL groups, no statistical differences in WTP values in all analyzed claims were found between normal weight people with good and poor body image (WTP^NH^, WTP^NL^). This result implies that the level of dissatisfaction of body image did not influence the WTPs for healthy potato chips among people of normal weight. On the other hand, the level of body image dissatisfaction affected obese people. In this regard, our results indicated that obese people with body image dissatisfaction were willing to pay a lower price for reduced-fat and low-salt potato chips than obese people with good body image (WTP^OH^, WTP^OL^). Conversely, when comparing normal weight and obese people with good body image, the obese people were willing to pay a higher price for reduced-fat and low-salt potato chips than normal weight consumers (WTP^NH^, WTP^OH^). Finally, when normal weight and obese people reported body image dissatisfaction, normal people were willing to pay for potato chips carrying both nutritional claims more than obese individuals. However, obese people were willing to pay a higher price for chips with a low-salt claim than normal weight people (WTP^NL^, WTP^OL^).

## 4. Discussion and Conclusions

The purpose of this study was to analyze the impact of the body image state of obese and non-obese individuals on purchase behavior using the Body Image Scale (BISS), proposed by Cash et al. [9]. Firstly, in this study, we demonstrated that obesity and body image dissatisfaction were highly positively correlated in the purchase behavior field, similarly to body image research in the eating behavior field. For example, the review of Schwartz and Brownell [37] reported that obese people were more dissatisfied with their body image than normal weight people. In the same line, Markey and Markey [38] showed that their heavier participants had the poorest body image out of the whole sample. 

Secondly, findings reported that body image state is heterogeneous in obese people. In other words, obese people reported both body image dissatisfaction and good body image. These findings are in line with Schwartz and Brownell [37] who found that although obesity is related to body image dissatisfaction, its level could be considerably heterogeneous among obese people. In the same line, the review conducted by Sarwer and Cash [39] indicated that not all studies have demonstrated that obese people were dissatisfied with their body image because of their body weight. For example, the authors reported the study carried out by Hill and Williams [40] which found the existence of a relationship between body image dissatisfaction and self-esteem and peer relationships rather than with BMI. 

Third, we demonstrated that body image dissatisfaction positively influenced the WTP values for healthy potato chips because obese individuals were willing to pay an extra price for potato chips carrying nutritional claims. This result is in accordance with Markey and Markey [38] and Contento et al. [41] who showed that non-clinical obese people with body image dissatisfaction made healthy eating changes. For example, Markey and Markey [38] reported that those obese people with body image dissatisfaction were more likely to begin a healthy diet. Likewise, Contento et al. [41] indicated that body image dissatisfaction of women influenced their dietary intake because they were accustomed to consuming food with low content of calories, fat, and sugar. Conversely, in our study, obese people with body image dissatisfaction were willing to pay a lower premium price for healthier chips than those obese individuals with good body image. This result is similar to Neumark-Sztainer et al. [42], who indicated that individuals with body image dissatisfaction were more concerned with being thinner rather than to have a healthy weight status. Therefore, a possible reason why obese people with body image dissatisfaction were willing to pay a premium price lower than those with good body image is because they were less worried about their health status. 

On the other hand, the findings demonstrated that consumers were willing to pay a higher price for potato chips with reduced-fat content than ones low in salt content, indicating that consumers made food decisions based on their beliefs that chips were healthier due to the low level of their fat content rather than their salt content. This may be because participants were less familiar with the nutritional claims related to salt content in the Spanish market [43].

The results of this study suggest that the body image dissatisfaction of obese people could impact their WTP for a bag of potato chips carrying health claims since their willingness to pay for healthier potato chips is lower than that of those who showed a good body image. Because a more negative body image carries a greater risk for body control behaviors in both obese and non-obese people, policy makers could design different education and outreach activities to increase knowledge about the risk of having a negative self-body image, in terms of eating disorders, extreme dieting, or extreme exercise compulsion. In this regard, workshops and activities targeting obese people with body image dissatisfaction could be promoted to increase their awareness about the negative consequences of presenting body image dissatisfaction, for example the increasing risk of disorders and non-communicable diseases (NCDs). Consequentially, obese people being more aware of their health status and less aware of beauty ideals could improve their perception of their body and thus, decide to make a conscious choice to exclude or reduce fat from their diet. Moreover, the improvement in perception of body image could be achieved if consumers increase their knowledge of nutrition, healthy eating habits, and healthy eating behavior. Nevertheless, in Spain there are still barriers among Spanish consumers in terms of understanding nutritional information, even if the public sector intervenes using the Nutrition, Physical Activity and Obesity Prevention (NAOS) strategy, which aims to promote healthy diet and boost physical activities to prevent the prevalence of obesity and consequently reduce the risk of NCDs.

These activities could be useful coping strategies in order to mitigate the negative impact of body image dissatisfaction of obese people on their WTP for healthy food products, to increase their willingness to pay a higher price for them. 

This study has some limitations due to the use of one scale to measure body image state, such as the BISS scale. The BISS scale measures body image state at a specific moment in time. Hence, further studies could apply the BISS scale at two different times, for example before and after the purchase or after a stimulus such as videos or images related to healthy habits. In the same line, future studies could include weight change and reason to control the weight gain/loss as a control variable to evaluate the main motivation for obese people to change their purchasing behavior. 

Finally, to give more robustness to our results, further studies are needed to confirm that BISS has an important role in consumer purchasing behavior in general and in obese consumers in particular. For example, it would be interesting to test the differences between WTP values in RCE and WTP values in another incentive compatibility method such as auctions also taking into account different mechanisms (e.g., random nth price auctions, BDM) or different target of consumers (obese people under medical treatment and those who are not). 

## Figures and Tables

**Table 1:** Attributes and levels used in the choice experiment design.

Attributes	Levels
Price	€0.50
	€0.95
	€1.40
	€1.85
Reduce-fat claim (Fat)	0 = No label
	1 = A reduced-fat chip is at least 30% less fat compared to traditional chips.
Low-salt content (Slt)	0 = No label
	1 = The amount of salt in the chips is not more than 0.03 g of salt per 150 g of product.

**Table 2:** Definition of socio-demographic variables of pooled sample and across the groups.

Variable Definition	Pooled Sample	NH ^a^	NL ^b^	OH ^c^	OL ^d^
	*n* = 309	*n* = 190	*n* = 61	*n* = 22	*n* = 36
Gender					
Male	40.1	36.1	17.2	53.9	38.2
Female	59.9	63.9	82.8	46.1	61.8
Age					
Between 18–35 years	28.5	40.5	41.4	12.1	23.5
Between 35–54 years	40.8	33.0	44.8	42.9	41.2
More than 54 years	30.7	26.5	13.8	45.0	35.3
Education of respondent					
Elementary School	19.7	13.2	6.9	33.0	19.1
High School	42.7	43.0	48.3	41.0	42.7
University	37.5	43.8	44.8	26.4	38.2

^a^ NH means normal weight with good body image; ^b^ NL means normal weight with body image dissatisfaction; ^c^ OH means obese people with good body image; ^d^ OL means obese people with poor body image dissatisfaction.

**Table 3:** Parameter estimates of Random Parameter Logit (RPL) for pooled sample and the four groups.

Mean Values	Pooled Sample	NH	NL	OH	OL
FAT	0.82	0.70	0.63	1.11	0.77
	(5.57) ***	(3.13) ***	(1.55)	(3.57) ***	(2.28) **
SALT	0.21	−0.16	−0.33	0.92	0.68
	(1.33)	(−0.66)	(−0.57)	(3.00) **	(2.04) **
FSALT	0.37	0.46	0.94	0.19	0.27
	(2.32) **	(1.88) *	(1.54)	−0.58	−0.77
NO BUY	−2.72	−3.06	−1.82	−2.39	−2.94
	(−15.32) ***	(−10.82) ***	(−3.60) ***	(−6.92) ***	(−7.39) ***
PRICE	−1.98	−2.14	−1.36	−1.61	−2.49
	(−19.68) ***	(−13.32) ***	(−4.69) ***	(−8.27) ***	(−10.92) ***
Standard deviations of parameter distributions					
FAT	1.91	1.76	1.55	2.67	2.08
	(13.64) ***	(7.88) ****	(3.94) ***	(6.89) ***	(5.28) ***
SALT	2.32	2.05	3.12	2.41	2.24
	(14.48) ***	(10.10) ***	(4.25) ***	(7.732) ***	(6.91) ***
FSALT	1.37	1.35	1.71	1.64	1.12
	(7.98) ***	(4.16) ***	(4.25) ***	(5.01) ***	(4.24) ***
χ^2^	2868.88	1068.13	198.96	958.05	698.62
n#obervations	11124	4356	1044	3276	2448
Pseudo R^2^	0.35	0.33	0.26	0.40	0.39
Loglikelihood	−2639.2	−1061.1	−282.8	−720.6	−547.1
H_0_ = Test of equality across sub − samples					54.9 ***

(***) (**) (*) denotes statistical significance at 1%, 5%, and 10% significance; z ratios in brackets.

**Table 4:** Statistical significance from Poe Test across four groups.

	FAT	SALT	FSALT
(WTP^NH^ − WTP^NL^) = 0	ns	ns	ns
(WTP^O^^H^ − WTP^O^^L^) = 0	**	**	ns
(WTP^NH^ − WTP^O^^H^) = 0	***	***	ns
(WTP^NL^ − WTP^O^^L^) = 0	ns	*	*

(ns) denotes no statistical difference; (***) (**) (*) denotes statistical significance at 1%, 5%, and 10% significance; WTP^NH^ means bootstrapped WTP estimates for normal weight with good body image; WTP^NL^ means bootstrapped WTP estimates for normal weight with body image dissatisfaction; WTP^OH^ means bootstrapped WTP estimates for obese people with good body image; WTP^OL^ means bootstrapped WTP estimates for obese people with body image dissatisfaction.
